# Increased Risky Choice and Reduced *CHRNB2* Expression in Adult Male Rats Exposed to Nicotine Vapor

**DOI:** 10.3390/ijms23031231

**Published:** 2022-01-22

**Authors:** Priscilla Giner, Liliana Maynez-Anchondo, Anna E. Liley, Kevin P. Uribe, Gabriel A. Frietze, Nicholas W. Simon, Ian A. Mendez

**Affiliations:** 1 Department of Psychology, The University of Texas at El Paso, El Paso, TX 79968, USA; pginer@utep.edu (P.G.); lmaynezanc@miners.utep.edu (L.M.-A.); kevinuribe21@gmail.com (K.P.U.); 2 Department of Psychology, The University of Memphis, Memphis, TN 38111, USA; anna.l@memphis.edu (A.E.L.); nwsimon@memphis.edu (N.W.S.); 3 Department of Pharmaceutical Sciences, School of Pharmacy, The University of Texas at El Paso, El Paso, TX 79968, USA; gafrietze@utep.edu

**Keywords:** nicotine, e-cigarette, risky choice, cholinergic, dopaminergic, rat

## Abstract

While the cognitive enhancing effects of nicotine use have been well documented, it has also been shown to impair decision making. The goal of this study was to determine if exposure to nicotine vapor increases risky decision making. The study also aims to investigate possible long-term effects of nicotine vapor exposure on the expression of genes coding for cholinergic and dopaminergic receptors in brain. Thirty-two adult male Sprague Dawley rats were exposed to 24 mg/mL nicotine vapor or vehicle control, immediately followed by testing in the probability discounting task for 10 consecutive days. Fifty-four days after the 10-day vapor exposure, animals were sacrificed and expression of genes coding for the α4 and β2 cholinergic receptor subunits, and dopamine D1 and D2 receptors, were analyzed using RT-PCR. Exposure to nicotine vapor caused an immediate and transient increase in risky choice. Analyses of gene expression identified significant reductions in *CHRNB2* and *DRD1* in the nucleus accumbens core and *CHRNB2* and *DRD2* in the medial prefrontal cortex of rats previously exposed to nicotine vapor, relative to vehicle controls. Results provide data on the negative cognitive effects of nicotine vapor exposure and identify cholinergic and dopaminergic mechanisms that may affected with repeated use.

## 1. Introduction

Successful commercial development of electronic cigarettes (commonly referred to as e-cigarettes) occurred in 2003. While they were originally developed as tool for smoking cessation, recreational use of e-cigarettes has increased at an alarming rate, particularly within the adolescent population [1]. Clinical studies investigating e-cigarette use have linked repeated nicotine vapor inhalation with negative health consequences that can differ from those produced from tobacco leaf smoke inhalation [2,3]. Preclinical studies have also begun to shed light on the toxic health effects of e-cigarettes, including the negative impact on the brain and behavior [4,5,6]. Research has shown that relative to tobacco leaf cigarettes, e-cigarettes expose users to distinct nicotine concentrations, chemical additives (e.g., salt, flavorings), and chemical reaction products (e.g., nicotyrine) that enhance nicotine’s rewarding properties and promote a transition to tobacco leaf cigarette use in individuals with no history of smoking [7,8,9,10].

Cognitive and behavioral deficits have been well documented in tobacco leaf cigarette smokers [11,12,13]. For example, smokers have been shown to exhibit increases in impulsive and risky choice compared to non-users [14,15,16]. Research is now beginning to identify similar decision-making deficits in e-cigarette users [17,18,19]. While clinical studies identify a link between nicotine exposure and poor decision making, they cannot address whether this cognitive deficit is pre-existing to nicotine use or occurs as a result of use. Animal studies examining changes in impulsive or risky choice following nicotine injections have reported mixed results. Several rodent studies have shown acute increases in impulsive choice following nicotine injections [20,21], while others have shown no effects [22,23] or decreases [24] in either impulsive or risky choice. Notably, when increases in impulsive choice were assessed after 65 days of repeated exposure to 0.3 or 1.0 mg/kg nicotine injections, increases in impulsive choice were seen up to 30 days after exposure [20]. It should be noted that nicotine injections in rats have been shown to increase perseverative behavioral responding in cost-benefit decision making tasks [22], an effect that could be contributing to the inconsistent findings reported in the literature. It was only recently that a study from our laboratory demonstrated that nicotine vapor inhalation causes short-term increases in impulsive choice, in rats [4].

A number of corticolimbic structures have been implicated in cost-benefit decision making, including the prefrontal cortex and striatum [25,26]. Experimental research on the neurobiological mechanisms underlying cost-benefit decision making in these regions has predominantly focused on the dopaminergic system [27,28], shedding light on how psychostimulant drugs of abuse drive poor decision making through modulation of dopaminergic signaling [29,30]. Investigation into the role of cholinergic signaling in decision making is less common, despite increased impulsivity and riskiness observed in smokers [16,31]. One animal study has linked low α4β2 nicotinic receptor expression in the nucleus accumbens and medial prefrontal cortex with decreased impulsive choice and increased risky choice, in naïve rats [32]. A more recent study found that complete knockout of β2 nicotinic receptor subunits in mice also resulted in increased risk behaviors [33]. These findings, linking low α4β2 levels with increased riskiness in naïve rodents is somewhat surprising, considering that nicotine exposure causes immediate upregulation of α4β2 nicotinic receptors in brain [34,35]. Additional work is needed to better characterize the cholinergic mechanisms driving decision making.

Nicotine-induced increases in risk-taking may promote ongoing substance use despite the possibility of negative consequences. The current rise in e-cigarette use highlights the need for additional preclinical research on the effects of nicotine vapor exposure on cost-benefit decision making and the mechanisms that underlie it. While nicotine vapor induced increases in impulsive choice have been demonstrated, the ability of nicotine vapor exposure to shift risky choice during uncertainty is not known. Thus, the goal of this study was to investigate the role of the cholinergic system in risky decision making by examining the ability of nicotine vapor exposure to shift choice preference in the probabilistic discounting task in adult male rats. Furthermore, to elucidate how nicotine use may affect cholinergic mechanisms in regions implicated in risky choice, the effects of nicotine vapor exposure on corticolimbic α4β2 nicotinic receptor subunit gene expression was also assessed.

## 2. Results

### 2.1. Training in the Probability Discounting Task

**Figure 1:**
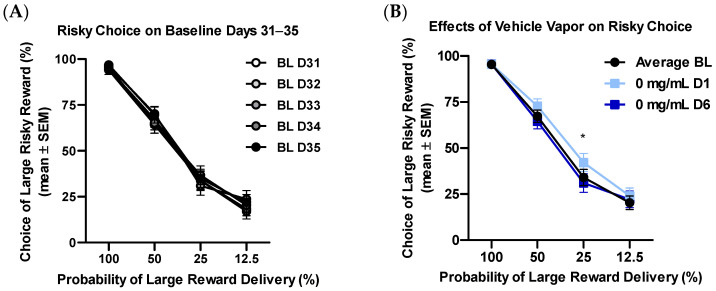
Stability in the probability discounting task. (**A**) Rats maintained stable risk curves across baseline (BL) training days 31–35. (**B**) An effect of vehicle vapor exposure was observed on day 1 of exposure. Importantly, this effect was no longer seen after 6 days of exposure to vehicle vapor. * *p* < 0.05 when comparing average BL to first day of exposure to vapor vehicle (0 mg/mL D1).

Rats were trained in the probability discounting task until criteria for stable responding were met (Training Days 31–35, Figure 1A). A mixed model ANOVA revealed a main effect of probability block (*F*_(3,90)_ = 274.76, *p* < 0.01, *η_p_*^2^ = 0.90), without a main effect of training day (*F*_(4,120)_ = 1.09, *p* = 0.37) or group (*F*_(1,30)_ = 0.01, *p* = 0.98). Additionally, no interactions involving probability block, training day, or group were observed (*F_s_* < 1.75, *p_s_* > 0.20). Preliminary studies in our laboratory have identified transient increases in risky choice preference following initial exposure to nicotine vehicle (unpublished); therefore, rats were habituated to vapor exposure procedures using nicotine vehicle for 6 consecutive days. As previously observed in our laboratory, a repeated measures ANOVA revealed a main effect of day (*F*_(1,31)_ = 9.97, *p* < 0.01, *η_p_*^2^ = 0.86) and a main effect of block (*F*_(3,93)_ = 246.79, *p* < 0.01, *η_p_*^2^ = 1.00), with no interaction of day and block (*F*_(3,93)_ = 2.02, *p* = 0.12), when comparing risky choice on nicotine vehicle exposure day 1 to average risky choice on training days 31–35 (Figure 1B). Post-hoc *t*-test comparing choice at each block revealed significant increases in risky choice on block 3 (12.5%, *p* < 0.01, Cohen’s *d* = 0.57), but not blocks 1, 2, or 4. Importantly, while a main effect of block was observed (*F*_(3,93)_ = 238.08, *p* < 0.01, *η_p_*^2^ = 1.00), no main effects or interactions of day were observed, when comparing risky choice after 6 days of vehicle vapor exposure to average risky choice during training days 31–35.

### 2.2. Effects of Nicotine Vapor Exposure on Risky Choice

To assess the effects of nicotine on rats’ ability to perform in the task, we compared trial omissions between the treatment groups on each of the 10 treatment days, using independent samples *t*-test. These analyses revealed no significant effect of nicotine on trial omissions on any of the 10 treatment days (*t_s_* < 1.16, *p_s_* > 0.26). The ability of nicotine vapor exposure to shift probabilistic discounting curves was assessed across all exposure days using a mixed model ANOVA. While a main effects of treatment day (*F*_(9,261)_ = 2.84, *p* < 0.01, *η_p_*^2^ = 0.96) and probability block (*F*_(3,87)_ = 225.08, *p* < 0.001, *η_p_*^2^ = 1.00) were observed, no main effect of vapor treatment group was seen (*F*_(1,29)_ = 1.28, *p* = 0.27). Additionally, no interactions between treatment day, probability block, or treatment group were observed (*F_s_* < 1.46, *p_s_* > 0.23).

**Figure 2:**
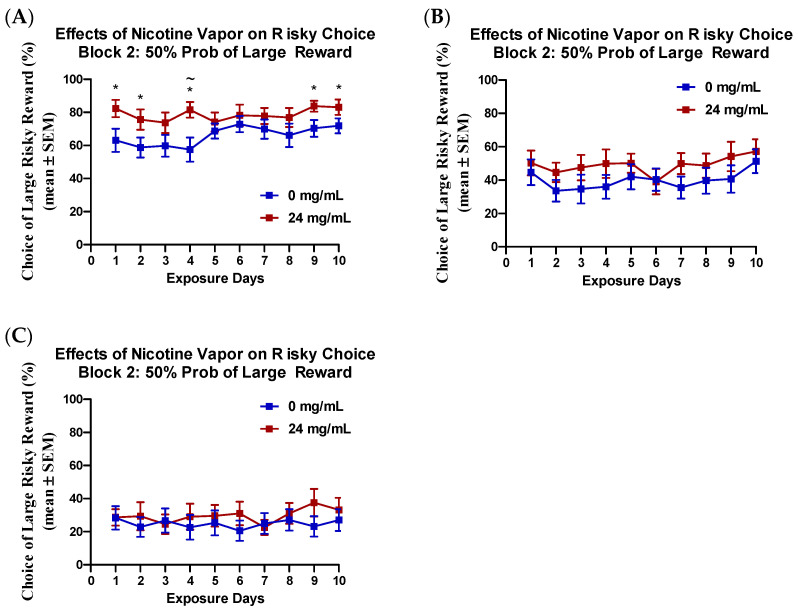
Effects of 24 mg/mL nicotine vapor exposure on risky choice preference. (**A**) Nicotine vapor exposure increased risky choice during block 2, when the large reward had a 50% probability of delivery, on days 1, 2, 4, 9, and 10. No effect of treatment was observed on risky choice during (**B**) block 3, when the large reward had a 25% probability of delivery or (**C**) block 4, when the large reward had a 12.5% probability of delivery. * *p* < 0.05; ~ Bonferroni correction < 0.005.

Previous work from our laboratory demonstrates that there is variability in the effects of nicotine on risky choice, between probability blocks [22,24]. To assess the effects of nicotine vapor exposure on each risky decision-making block exclusively, a priori mixed model ANOVAs, with subsequent *t*-test post-hoc analyses, were conducted across treatment days, for each probability block where risk of reward loss could occur (blocks 2, 3, and 4). Analysis of choice preference between groups and across treatment days for probability block 2 (50% probability of delivery of larger risky reward) revealed a main effects of treatment day (*F*_(6,270)_ = 2.07, *p* < 0.05, *η_p_*^2^ = 0.86, Figure 2A) and a main effect of treatment group (*F*_(1,30)_ = 4.13, *p* = 0.05, *η_p_*^2^ = 1.00). No interaction of treatment day and vapor treatment group was seen for block 2 choice preference. Additionally, no main effects or interactions of treatment day or group were seen for probability block 3 (25% probability of delivery, Figure 2B) or probability block 4 (12.5% probability of delivery, Figure 2C). Subsequent *t*-test comparing risky choice between treatment groups on block 2, during each individual treatment day, revealed significant increases in risky choice on vapor exposure days 1, 2, 4, 9, 10 (*t_s_* > 1.75, *p_s_* < 0.05, Cohen’s *d* > 0.62, Figure 2A). When testing risky choice in the probability discounting task on the 3 days immediately following 10-days of nicotine vapor exposure, a main effect of block was observed on each day (*F_s_* > 105.84, *p_s_* < 0.01, *η_p_*^2^*_s_* = 1.00), but no main effects or interactions with vapor treatment group were detected (*F_s_* < 0.72, *p_s_* > 0.54).

### 2.3. Effects of Nicotine Vapor Exposure on Perseverative Responding

The ability of acute nicotine vapor exposure to cause perseverative lever pressing was assessed using a mixed model ANOVA. Analysis shows that while there was a main effect of block (*F*_(3,63)_ = 633.54, *p* < 0.01, *η_p_*^2^ = 1.00), no main effect of group (*F*_(1,21)_ = 0.07, *p* = 0.80) or interaction of group and block (*F*_(3,63)_ = 0.06, *p* = 0.98) was observed (data not shown).

### 2.4. Effects of Nicotine Vapor Exposure on Gene Expression

**Figure 3:**
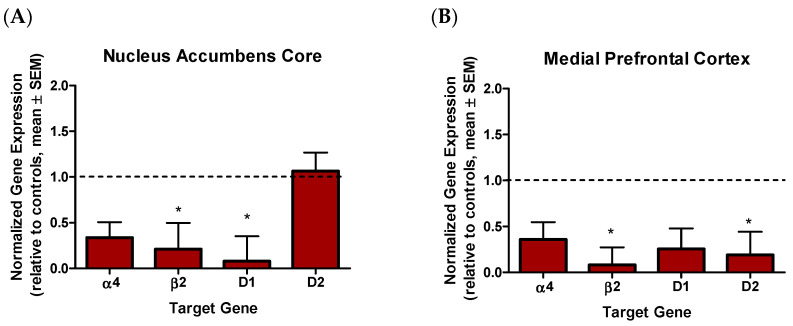
Effects of 24 mg/mL nicotine vapor exposure on gene expression, normalized and relative to controls. (**A**) Significant reductions in gene expression were seen for *CHRNB2* and *DRD1* genes in nucleus accumbens core of rats previously exposed to nicotine vapor, relative to controls. (**B**) Significant reductions in gene expression were seen for *CHRNB2* and *DRD2* genes in medial prefrontal cortex of rats previously exposed to nicotine vapor, relative to controls. * *p* < 0.05.

Nine days after the perseverative responding test, and 54 days following chronic nicotine vapor exposure, a subset of animals used for behavioral testing were sacrificed and brains were collected. Nicotinic and dopaminergic receptor gene expression in the nucleus accumbens core and shell, the medial prefrontal cortex, and the orbitofrontal cortex, was investigated. While independent samples *t*-test revealed no effect of treatment on *CHRNA4* expression in any of the brain regions assessed (*t_s_* < −0.91, *p_s_* > 0.39), a significant reduction in *CHRNB2* expression was observed in the nucleus accumbens core (*t*_(6)_ = −2.42, *p* = 0.05, Cohen’s *d* = −1.71, Figure 3A) and medial prefrontal cortex (*t*_(9)_ = −2.94, *p* < 0.05, Cohen’s *d* = −1.78, Figure 3B), but not nucleus accumbens shell or orbital frontal cortex (*t_s_* < −1.11, *p_s_* > 0.29) of animals previously exposed to 24 mg/mL nicotine, relative to rats exposed to vehicle vapor. Interestingly, a reduction in *DRD1* expression was also seen in the nucleus accumbens core (*t*_(6)_ = −3.78, *p* < 0.01, Cohen’s *d* = −2.67) and a reduction in *DRD2* expression was seen in the medial prefrontal cortex (*t*_(9)_ = −2.95, *p* < 0.05, Cohen’s *d* = 1.79) of rats previously exposed to 24 mg/mL nicotine vapor, when compared to rats previously exposed to vehicle vapor. No other effects of dopamine receptor gene expression were observed (*t_s_* < −1.96, *p_s_* > 0.08). 

## 3. Discussion

The goal of the present study was to examine the ability of nicotine vapor exposure to shift risky choice, as determined by the probabilistic discounting task, in adult male rats. In an attempt to model human e-cigarette use, rodents were passively exposed to nicotine vapor using a four-chamber passive vapor inhalation system. Our findings demonstrate that exposure to 24 mg/mL nicotine vapor causes transient increases in risky choice, similar to that previously reported for impulsive choice [4]. Following behavioral testing, nicotinic and dopaminergic receptor gene expression was assessed in corticolimbic structures of rats previously exposed to nicotine vapor or vehicle control. Interestingly, rats with extended nicotine vapor exposure exhibited reduced *CHRNB2* and *DRD1* expression in the nucleus accumbens core and reduced *CHRNB2* and *DRD2* expression in the medial prefrontal cortex, relative to vehicle controls.

The probability discounting task is a validated behavioral task that has been used successfully in our laboratory, and others, to investigate choice preference for larger rewards with decreased probability of delivery [22,25]. This risk of losing reward is similar to other “gambling” tasks used in rats and was selected due to extensive use in our laboratory, and others, as well as its ability to assess various probabilities of reward delivery within a single test session. Early exploratory studies in our laboratory revealed that initial 90 min exposure sessions to vehicle vapor shifted select behavioral measures, including discounting curves in the probabilistic discounting task. In the present experiment, increases in risky choice were again seen after the first day of vehicle vapor exposure (Figure 1B). Importantly, when testing risky choice after 5 additional days of vehicle vapor exposure, discounting curves were found to return baseline levels. The observed increase in risky choice seen following day 1 of vehicle vapor exposure may be driven by a number of variables, including stress from the initial passive vapor exposure session or the even the sweet taste of vegetable glycerin. The ability of stress to induce increases in riskiness has been well documented [see reference [36] for review] and relative to self-administration, passive exposure to drugs has indeed been shown increase stress hormone levels in rodents [37]. Similarly, increased sensitivity to sweet tastants has been associated with increases in impulsivity that can affect behavioral responding. These findings suggest that pre-exposure to vehicle vapor when using passive exposure procedures should be considered for future studies investigating the effects of nicotine vapor on decision making.

The probability discounting task was used to assess risky decision making. Exposure to 24 mg/mL of nicotine vapor was found to cause modest, short-term increases in risky choice. When comparing choice preference during the 2nd block (when the large reward had a 50/50 chance of being delivered), rats exposed to nicotine vapor showed a significant increase in risky choice, on 5 of the 10 nicotine vapor exposure days (Figure 2A). On the 3 days immediately following repeated nicotine vapor exposure, no differences in risky choice were observed between the nicotine exposed rats and controls. Additionally, a test of behavioral perseveration immediately following 24 mg/mL nicotine vapor exposure showed that increases in risky choice were not due to reduced behavioral flexibility. The ability of nicotine vapor exposure to cause immediate, short-term increase in risky choice is similar to the nicotine vapor-induced increase in impulsive choice previously reported by our laboratory [4]. While group differences during the 50/50 block only reached statistical significance on half of the nicotine vapor exposure days, a trend for increased riskiness appears on all exposure days. A lack of effect observed on exposure days 5–8 appears to be driven by a slight increase in risky choice in the vehicle control group. While the cause for this slight shift in the control group may be due to day-to-day variability in the task, it should be noted that the behavioral effects of repeated exposure to vehicle vapor alone remain unclear. Increases in risky choice following exposure to vehicle vapor were observed following initial exposure, and it remains possible that these effects also manifest later with repeated exposure to vehicle vapor. On the days where significant group differences were seen, it remains possible that longer exposure to nicotine vapor may result in a more potent and/or persistent increases in risky choice, as previous studies using nicotine injections observed long-lasting effects of nicotine on impulsive choice, after 65 days of nicotine treatment. Additional studies with larger treatment groups and extended nicotine vapor exposure will be needed to further characterize the nature of nicotine’s subtle effects on risky and impulsive choice.

Previous research in our laboratory has demonstrated that exposure to 24 mg/mL nicotine vapor, using the described exposure parameters in the current study, results in plasma cotinine levels (a metabolite of nicotine) that are within the range of those commonly seen in daily smokers and e-cigarette users, as well as following injections of nicotine in rodents (100–300 ng/mL) [4,38,39,40]. Studies from other laboratories examining cotinine levels following exposure to lower concentrations of nicotine vapor in mice (6 mg/mL) and rats (5 mg/mL) have observed lower cotinine levels in the range of 20 to 70 ng/mL when using free base nicotine liquids, as we did in our study [10,41]. Importantly, these studies also identify other variables requiring consideration when determining vapor exposure parameters. In the mouse study, lower cotinine levels following exposure to the vapor of a freebase nicotine solution versus the vapor of a salt nicotine solution, were observed [10]. In the rat study, lower cotinine levels were seen in females versus males exposed to the same concentration of nicotine vapor. While cotinine levels following nicotine vapor exposure have been well studied, investigations also now showing that rats will self-administer various concentrations of nicotine vapor (0, 0.05, 0.5, 5, 50 mg/mL) in a dose dependent manner [42], suggesting that our concentration of nicotine vapor is reinforcing. Finally, a recent study in our laboratory identified significant increases in not only cotinine levels, but also physical withdrawal signs, anxiety-like behavior, and intracranial self-stimulation thresholds, following precipitated withdrawal from repeated nicotine vapor exposure using the same parameters described in this study [see reference [43] for preprint describing this work]. Together these findings substantiate our exposure procedures and delivery system as an effective model of human e-cigarette use.

The modest and transient shifts in impulsive and risky choice observed following nicotine vapor exposure may explain why the effects of nicotine on cost-benefit decision making have not always been reported in pre-clinical studies. A number of procedural and biological factors must be considered by future studies investigating the effects of nicotine vapor on decision making, and animal behavior in general. For example, subsequent research investigating the effects of nicotine vapor exposure on behavior should consider analysis of both plasma cotinine and nicotine levels during testing. Extended daily exposure to nicotine vapor should also be considered, as it may yield more robust nicotine dependence and intake, similar to that seen with extended access to nicotine intravenous self-administration [44]. Differences in nicotine withdrawal following repeated nicotine injections have been observed when comparing males and females, adolescents and adults, and various strains in rodents [45,46,47]. Therefore, it remains possible that the observed modest effect of nicotine vapor on risky choice was specific to the adult male Sprague Dawley rats used in this study. Further investigation on how factors such as sex, age, and species strain impact the effects of nicotine vapor exposure on decision making is necessary. 

In addition to the effects of nicotine vapor on risky choice, our study examined *CHRNA4*, *CHRNB2*, *DRD1*, and *DRD2* expression in the corticolimbic structures of rats with and without a history of repeated nicotine vapor exposure. The prefrontal cortex and striatum were investigated, as these regions have been heavily implicated in cost-benefit decision making [25,26,28]. While the literature on the effects of e-cigarette use on cholinergic mechanisms is very limited, human studies have recently begun to identify changes in the expression of non-cholinergic genes [48,49] and the function of β2 cholinergic receptors [50] of e-cigarette users. Previous pre-clinical studies examining nicotinic receptor protein levels have focused extensively on changes observed soon after exposure to nicotine injections or minipumps (within 24 h), and repeatedly show that this exposure causes upregulation of α4β2 receptors in mesolimbic and basal forebrain cholinergic systems [51], an effect that has been reported to persist for about 1 month after exposure [52,53]. Pre-clinical research on the effects of nicotine vapor on cholinergic receptors is also limited, but has shown that exposure to 24 mg/mL nicotine vapor, as well as flavorants used in nicotine solutions, can affect nicotinic receptors levels and function in the striatum [54]. Immediate effects of nicotine injections on *CHRNA4* and *CHRNB2* expression has been limited compared to receptor analysis, with some studies showing no changes in nicotinic receptor subunit gene expression [55,56] and others showing immediate increases in both *CHRNA4* and *CHRNB2* expression, similar to that seen with α4β2 receptors [57,58]. To our knowledge, there is no pre-clinical work on the effects of nicotine vapor exposure on cholinergic receptor gene expression.

In this experiment, we examined nicotinic and dopaminergic receptor gene expression 54 days after repeated nicotine vapor exposure. Our findings revealed reduced expression of *CHRNB2* and *DRD1* in the nucleus accumbens core, as well as reduced expression of *CHRNB2* and *DRD2* in the medial prefrontal cortex, of animals previously exposed to nicotine relative to those exposed to vehicle vapor (Figure 3). Dopamine activity in corticolimbic structures has been implicated in risky choice [59,60]. For example, one study by Simon et al. (2011) demonstrated that *DRD1* but not *DRD2* expression in the nucleus accumbens predicted choice in a punishment risk discounting task, while *DRD2* but not *DRD1* expression in the medial prefrontal cortex predicted choice in this same task. Interestingly, in our analysis we found that relative to controls, animals previously exposed to nicotine vapor exhibited reductions in *DRD1,* but not *DRD2,* in the nucleus accumbens core and reductions in *DRD2,* but not *DRD1,* in the medial prefrontal cortex.

Only a handful of studies have examined the long-term effects of nicotine exposure on nicotinic subunit gene expression and findings have been mixed, with one study reporting no changes in gene expression 180 days after adolescent exposure [61] and another showing reduced expression of *CHRNB2* in the prefrontal cortex 4 months after postnatal exposure [62]. One other study identified reduced expression of *CHRNB2* in prefrontal cortex following nicotine exposure in male rats [63]; however, the time between nicotine exposure and tissue collection was unclear. Interestingly, the latter study also presented sex differences in nicotinic receptor gene expression, with females showing reduced expression of both *CHRNA4* and *CHRNB2* in the prefrontal cortex. Future studies investigating the long-term effects of nicotine on both cholinergic genes and receptor levels are challenging, however, as they must consider other factors that can influence gene expression in the treatment group, such as nicotine withdrawal. Careful measurements of nicotine withdrawal are needed to determine if it is a moderating variable in any relationships that may arise between decision making and gene expression. Short-term increases in nicotinic receptor gene expression, followed by long-term decreases in the expression of these same genes, may suggest distinct, time-dependent changes in cholinergic mechanisms during abstinence. *CHRNA4* and *CHRNB2* are also highly implicated in nicotine reinforcement, as demonstrated with pre-clinical self-administration studies [64,65]. Therefore, time-dependent changes in cholinergic gene expression could underlie unique behavioral and cognitive processes driving relapse that occurs days, versus months after last nicotine use. Overall, these findings highlight the need for additional work on how nicotine vapor exposure affects cholinergic mechanisms and how these changes impact decision making.

## 4. Materials and Methods

### 4.1. Subjects

Thirty-two male Sprague-Dawley rats were obtained at 3 weeks of age from an outbred stock of animals (Envigo, Inc., Indianapolis, IN, USA). The rats were paired-housed in a humidity- and temperature-controlled (22 °C) vivarium on a reverse 12-h light/dark cycle (lights off at 6:00 a.m. and on at 6:00 p.m.) with ad libitum access to food and water, except as noted below. All animal procedures were approved and performed in accordance with the University of Texas at El Paso Animal Care and Use Committee’s regulations.

### 4.2. Experimental Design

**Figure 4:**
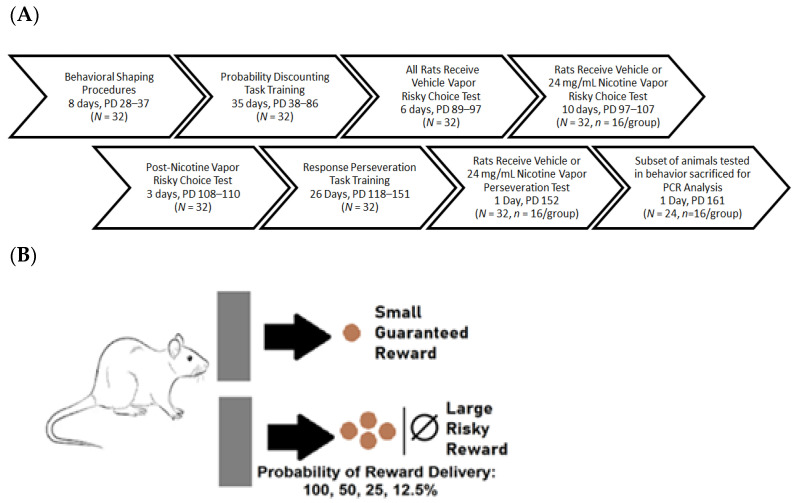
Experimental Design. Schematics depicting (**A**) timeline for experimental procedures and (**B**) structure of probability discounting task. Task requires rat to choose between two levers. A press on one lever results in delivery of a small food reward with guaranteed delivery. A press on the other lever results in delivery of a large food reward with probability of delivery.

Prior to the start of the experiment, the rats were handled in the vivarium for two consecutive days and food deprived to 90% of their free feeding weight, where they were maintained during behavioral training and testing. The experiment began with shaping procedures for the probability discounting task (see reference [4] for detailed description of shaping procedures). Shaping procedures were conducted until a criterion of 30 responses in 1 h was met by all rats, for each procedure. After shaping was complete, all rats were trained once daily in the probability discounting task, until a stable probabilistic discounting curve was achieved in the task (see Section 4.7 for description of stability criteria). All rats were then habituated once daily to passive vapor exposure procedures using nicotine vehicle (50% propylene glycol/50% vegetable glycerin), with each exposure session followed immediately by training in the probability discounting task. After 6 habituation days, rats were divided into two groups (24 mg of free base nicotine in 1 mL of vehicle or vehicle control, *n* = 16/group) and exposed to vapor for ten consecutive days. To examine the effects of nicotine vapor exposure on risky choice, all animals were tested in the probability discounting task immediately following vehicle or 24 mg/mL nicotine vapor exposure sessions. Choice preference without nicotine vapor exposure was assessed for three consecutive days after the last vapor exposure session. To investigate possible behavioral perseveration effects of 24 mg/mL nicotine vapor all rats tested in the probability discounting task were trained in a perseveration task until stable and the effects of nicotine vapor exposure were assessed. Ten days after the perseveration test, and 54 days after chronic nicotine vapor exposure, a subset of rats used for behavioral testing (*N* = 24, *n* = 12/group) were sacrificed and brains were collected. Brain tissue from the nucleus accumbens and prefrontal cortex were analyzed for nicotinic and dopaminergic receptor gene expression using RT-PCR (see Figure 4A for schematic of experimental timeline).

### 4.3. Nicotine Vapor Exposure

Rats received passive nicotine vapor exposure using the Four Chamber Benchtop Passive E-Vape Inhalation System (La Jolla Alcohol Research Inc., La Jolla, CA, USA). This system consisted of four sealed chambers (interior dimension of 14.5′′ L × 10.5′′ W × 9.0′′ H), each with two valve ports on opposing walls. One valve port was connected to a small vacuum that controlled the airflow in the chamber at 0.6 L per minute. The vacuum outlet was connected to a Whatman HEPA filter (Millipore Sigma, Darmstadt, Germany) and onto a house exhaust that safely removed the nicotine vapor from the chambers and testing room. The other valve port was connected via PVC tubing to a modified TFV4 mini-tank (4.9-volt, 65.0 W; Smok Inc., Shenzhen, China), where the nicotine was heated using 0.42 Ω coils. The mini tanks were also connected to a heating control system.

The nicotine vapor solution concentration and vapor exposure protocols with rodents were based on established parameters used to model human nicotine consumption rates and serum cotinine levels resulting from daily e-cigarette use [66,67]. For detailed description of vapor exposure procedures and additional data on the ability of our exposure parameters to produce cotinine levels and dependence in rodents, similar to that seen in human nicotine users or following injections of nicotine, see previous work from our laboratory [4] and preprint [43]. Briefly, rats were exposed to vapor in daily 90-min sessions consisting of 4 cycles, with 5-min inter-cycle-intervals. For each cycle, nicotine solution was heated to 400 degrees Fahrenheit for a 3 s puff delivery, occurring every 2 min and 10 times per cycle, for total of 40 puff deliveries per day and a cycle duration of 18 min and 37 s. For the present study, we utilized pre-made commercial flavorless nicotine e-liquids purchased from Vapor Vapes Inc. (Sand City, CA, USA).

### 4.4. Probability Discounting Task

To assess risky choice the study utilized eight standard operant chambers (Med Associates, Fairfax, VT, USA), where rats were trained and tested in a probability discounting task. Probability discounting sessions consisted of 4 blocks, with 10 risky or “safe” food reward choice trials per block. On each trial, a nose poke into the food trough extinguished the food trough light and triggered extension of either a single lever (forced choice trials) or both levers simultaneously (free choice trials). A press on one lever (either left or right) resulted in the delivery of a single food pellets (a small, safe reward). A press on the other lever resulted in the delivery of four food pellets (a large, risky reward). Failures to press either lever were scored as omissions. During the first block of trials, the probability of large reward delivery was 100%. During each of the next three blocks, the probability of large reward delivery decreased (100, 50, 25, and 12.5%). All left and right lever reward assignments and presentations were randomly assigned and counterbalanced across rats during all training and testing sessions (see Figure 4B for schematic of probability discounting task).

### 4.5. Perseverative Responding Task

Perseverative responding was also assessed in the operant chambers, with training beginning immediately after testing in the probability discounting task. Perseverative responding sessions were identical to those described for the probability discounting task, except in this case the large reward delivery was 100% guaranteed for blocks 1 and 2 and completely omitted for blocks 3 and 4. All left and right lever reward assignments and presentations were randomly assigned and counterbalanced across rats during all training and testing sessions for the perseveration task. Rats were trained in the task until stable responding was achieved (as described for training in the probability task). Once stable, rats were exposed to vehicle or 24 mg/mL nicotine vapor (receiving the same treatment administered during chronic vapor exposure) and tested in the perseverative responding task.

### 4.6. Gene Expression

RT-PCR was used to assess mRNA levels, with inferences about target gene expression based on analyses of associated mRNA. A subset of 24 rats from the 32 rats used for behavioral testing (12 per treatment group) were used for analysis of gene expression. Rats were decapitated, brains were collected, flash-frozen, and stored in a −80 °C freezer. Tissue samples were sliced with the cryostat Leica CM1860 (Leica Biosystems, Nussloch, Germany) and punched from the nucleus accumbens core, nucleus accumbens shell, the medial prefrontal cortex, and the orbitofrontal cortex using a 1 mm micro-punch pen (Electron Microscopy Sciences, Hatfield, PA, USA). Punches from both hemispheres were pooled. Total RNA was isolated using an Ambion^®^ RNAqueous^®^-Micro kit. RNA was then digested with DNAse I (Invitrogen, Waltham, MA, USA) prior to cDNA synthesis to remove any DNA contamination. Following the DNAse I digestion, RNA was quantified using a BioPhotometer^®^ D30 (Eppendorf, Enfield, CT, USA). The inclusion criterion for all RNA samples was a A260/280 purity ratio of 1.8 and above. RNA samples were reverse transcribed into cDNA with the AdvantageRT-for-PCR kit (Clontech, Mountain View, CA, USA) using Oligo(dT) primers, for cDNA synthetization. The samples were then diluted 1:10 in nuclease-free water and stored at −20 °C. Specific primers for the following genes were obtained from Integrated DNA Technologies Inc. (Coralville, IA, USA): α4 nicotinic receptor subunit (*CHRNA4*), β2 nicotinic receptor subunit (*CHRNB2*), D1 dopamine receptor (*DRD1*), D2 dopamine receptor (*DRD2*), and the common reference gene *GAPDH*. *GAPDH* was selected as our reference gene based on preliminary work from our laboratory, and work from others, suggesting that it is both stable and reliable for analysis of our target genes in adult male rats [67]. Ct value for target genes were normalized for individual rats by subtracting the average Ct value for a given target gene from the average Ct values for the references gene, in each region of interest. The cDNA primer sequences are illustrated in Table 1.

### 4.7. Statistical Analysis

Criterion for behavioral stability and choice preference in the probability discounting task was compared using mixed model analysis of variance (ANOVA) with group (vehicle control or 24 mg/mL nicotine) as a between-subject factor and reward probability block and training day as within-subject factors. Criterion for stability in the discounting and perseveration task was defined as a main effect of session block, with no main effect of day across 5 consecutive days. Gene expression in vehicle and nicotine vapor exposed rats were compared using independent squared *t*-tests. Bonferroni corrections were applied to each family of *t*-test analysis. Finally, partial eta squared and Cohen’s *d* were calculated to determine effect sizes.

## 5. Conclusions

Findings from this study provide much need data on the effects of nicotine vapor exposure on risky decision making and the mechanisms that drive choice. Behavioral data demonstrates modest and transient effects of nicotine vapor on decision making, an effect that is supported by other studies investigating cost-benefit decision making [4,21]. Research demonstrating the negative behavioral and cognitive effects of nicotine vapor exposure is imperative, as misconceptions about the safety of nicotine vapor has been identified as a factor driving recent increases in recreational e-cigarette use [68]. Our analysis also serves to extend the current understanding of nicotine’s possible effects on mechanisms underlying decision making. The observed reduction in nicotinic receptor subunit gene expression observed in subjects with a history of vapor exposure, versus those without, may be a distinct long-term mechanism driving relapse in e-cigarette users.

## Figures and Tables

**Table 1:** cDNA primer sequences for utilized genes.

Gene	Forward Primer Sequence (5’ to 3’)	Reverse Primer Sequence (3’ to 5’)
* GAPDH	CAA CTC CCT CAA GAT TGT CAG CAA	GGC ATG GAC TGT GGT CAT GA
*CHRNA4*	CAA CTT TCT GCA ACC CCA CG	TGG CAA CGT ATT TCC AGT CC
*CHRNB2*	AAG TGA GCG ACA CTG GTC TG	GTC TGG AGC CCT CTG AGG TA
*DRD1*	GAC ACA AGG TTG AGC A	CTG GGC AAT CCT GTA GAT A
*DRD2*	GTG TGT TCA TCA TCT GCT	GAA CTC GAT GTT GAA GGT

* Reference gene.

## Data Availability

Data is contained within the article. Additional details available upon request.
